# Investigation on birth weight outcomes in schistosomiasis and praziquantel research: a correspondence

**DOI:** 10.1186/s40001-023-01202-7

**Published:** 2023-07-12

**Authors:** Martha C. Holtfreter, Johannes Mischlinger, Saskia Dede Davi, Benjamin T. Schleenvoigt

**Affiliations:** 1 Tropical Medicine Unit, Department of Gastroenterology, Hepatology and Infectious Diseases, Faculty of Medicine, Heinrich-Heine-University, Moorenstr. 5, 40225 Düsseldorf, Germany; 2 Centre of Tropical Medicine, Bernhard Nocht Institute for Tropical Medicine & I. Department of Medicine University Medical Center Hamburg-Eppendorf, Hamburg, Germany; 3 German Centre for Infection Research (DZIF), partner site Hamburg-Luebeck-Borstel, Hamburg, Germany; 4 Institute of Infectious Diseases and Infection Control, Jena University Hospital, Am Klinikum 1, 07747 Jena, Germany

## Abstract

Infection with *Schistosoma* sp. during pregnancy can cause low birth weight of the newborn. To allow a better differentiation between newborns with low birth weight and those with normal weight, the terms of intrauterine growth restriction (IUGR), small for gestational age (SGA) or fetal growth restriction (FGR) should be used. FGR describes the relationship between birth weight and gestational age and is defined as the incapability of a fetus to achieve expected growth with birth weight below the 10th percentile for gestational age. Additional investigations of the proportion of newborns with FGR should obtain more certainty about the effect of praziquantel and schistosomiasis on fetal growth.

With great interest we read the article “Efficacy and safety of praziquantel for the treatment of human schistosomiasis during pregnancy: a phase 2, randomised, double-blind, placebo-controlled trial” published by Olveda and colleagues [1]. In addition to this work, only one other randomised controlled trial regarding the side effects of praziquantel during pregnancy is available in recent literature [2]. Both studies showed that praziquantel was safe and had no significant effect on birth weight. Although WHO recommends treatment of schistosomiasis during pregnancy, many countries remain reluctant to follow this approach [3].

The study conducted by Ndibazza and colleagues recruited 2507 pregnant women and investigated the effect of praziquantel given in the second and third trimester [2]. Overall low birth weight (< 2500 g) occurred in 8% (9·5% vs. 8·6% in placebo and praziquantel study arms, respectively) and there was no effect of praziquantel on the percentage of newborns with low birth weight (praziquantel vs. placebo OR: 0·96 (95% CI 0·70–1·32).

Olveda and colleagues investigated 370 pregnant women who were positive for *Schistosoma japonicum* [1]. The study group was given praziquantel in the second trimester of pregnancy and birth weight outcomes were compared with controls who received placebo. Low birth weight (< 2500 g) was observed in 12·7% vs. 16·1% in placebo and praziquantel study arms, respectively, and the difference was statistically not significant (*p* = 0.35). Notably, Olveda and colleagues evaluated the ratio of birth weight to the gestational age of newborns and reported the proportion of births that were too small for gestational age (SGA) separately. There was no difference in the frequency of SGA and the percentage of newborns in the study- and control group was 26·6% vs. 23·7%, respectively (*p* = 0·52).

The study data of Olveda and colleagues and Ndibazza and colleagues show that praziquantel has no negative effect on intrauterine growth when administered in the second and third trimester of pregnancy [1, 2].

A similar approach was used by Qunhua and colleagues and Mombo-Ngoma and colleagues for the investigation of adverse effects of schistosomiasis on pregnancy [4, 5]. Qunhua and colleagues analyzed the data of 244 pregnant women infected with *Schistosoma japonicum* compared to a healthy control group [4]. The birth weight of first newborns with *S. japonicum* infected mothers was significant lower than for newborns in the control group (3229 g vs. 3355 g, respectively; *p* < 0·05).

In the study of Mombo-Ngoma and colleagues, the percentage of newborns with low birth weight (< 2500 g) and premature delivery (< 37 weeks) was examined in *S. haematobium* infected women [5]. Mean birth weight in the schistosomiasis positive group was observed to be lower (2875·9 g/ 95% CI 2747·4–3004·4) than in the schistosomiasis negative group (2956·6 g/95%CI 2923·0–2990·2). The risk for low birth weight in the offspring of mothers with schistosomiasis was significantly increased (OR: 1·7; *p* = 0·04), whereas the risk for preterm delivery (< 37 weeks) seemed to be unaffected (OR: 1·07; *p* = 0·84).

The authors conclude that the weight of the first newborn to women with schistosomiasis is lower and that these pregnant women are at higher risk for low birth weight deliveries [4, 5].

From our point of view, the study endpoints “birthweight” and “low birth weight” should be used with caution, because birthweight depends on the gestational age. To allow for a better differentiation between newborns with low birth weight and those with normal weight, we would like to propose the terms of intrauterine growth restriction (IUGR), small for gestational age (SGA) or fetal growth restriction (FGR) that are frequently used synonymously as it was already done by Olveda and colleagues. FGR describes the relationship between birth weight and gestational age. It is defined as the incapability of a fetus to achieve expected growth with birth weight below the 10th percentile for gestational age [6, 7]. It normally occurs in 5–10% of all pregnancies and is comparable across multi-ethnic populations for newborn boys and girls [8, 9].

To obtain more certainty about the effect of praziquantel and urogenital schistosomiasis on fetal growth from the studies published by Ndibazza and colleagues and Mombo-Ngoma and colleagues, we would like to propose the additional investigation on the proportion of newborns with FGR from the available data of birth weight and gestational age separately for boys and girls. Regarding the data published by Olveda and colleagues, we would also like to suggest the examination of fetal sex in its potential influence on SGA to rule out a sex specific effect of praziquantel on intrauterine growth.

## Data Availability

Not applicable.
